# The prevalence and causes of visual impairment in special schools and blind schools in Shanxi Province, China: a cross-sectional study

**DOI:** 10.1038/s41598-025-07364-0

**Published:** 2025-07-11

**Authors:** Fengchao Zhou, Tianfeng Feng, Ge Bai, Congcong Linghu, Xiaowei Zhang, Hui Dong, Yan Zhou, Yuxia Zhao, Jianlin Zeng, Lihua Zhang

**Affiliations:** 1 Beijing Tongren Eye Center, Beijing Tongren Hospital, Capital Medical University, Beijing, 100730 China; 2 Department of Optometry, Shanxi Eye Hospital Affiliated to Shanxi Medical University, 100 Fudong Street, Xinghualing District, Taiyuan City, 030002 Shanxi Province China; 3 Department of Community Eye Care, Shanxi Eye Hospital Affiliated to Shanxi Medical University, 100 Fudong Street, Xinghualing District, Taiyuan City, 030002 Shanxi Province China

**Keywords:** Low vision, Blindness, Special school, Blind school, Paediatric research, Eye abnormalities

## Abstract

This study aimed to determine the prevalence and causes of low vision and blindness among students in special education schools (serving children with disabilities, including intellectual disabilities) and blind schools (for students with blindness or visual impairment) in Shanxi Province, China. A cross-sectional investigation was conducted across nine special education schools and two blind schools in Shanxi Province from March to September 2018. Participants underwent comprehensive ophthalmic examinations, including visual acuity (VA) assessment using Lea Symbols or Cardiff Cards, refraction testing, and anterior/posterior segment evaluations. Non-cycloplegic refraction was performed initially, with cycloplegic refraction administered when VA was subnormal for age. Visual impairment was classified according to World Health Organization (WHO) criteria. Demographic and general health data were also collected. The study included 441 children from special schools (mean age: 12.3 ± 3.5 years) and 56 children from blind schools or blind classes within special schools (mean age: 13.0 ± 3.2 years). Among special school students, 44 (10.0%) had visual impairment (mild: 9.1%; moderate: 0.9%), primarily attributable to uncorrected refractive errors (90.7%); 10 cases (2.3%) remained undetermined due to poor compliance. In blind schools/classes, 30 children (53.6%) were classified as blind, 22 (39.3%) as visually impaired (moderate: 28.6%; severe: 8.9%), and 4 (7.1%) as undetermined. Notably, 44.6% of visual impairment cases were avoidable, with nystagmus (25.0%), amblyopia (19.6%), and cataract (8.9%) identified as leading causes. These findings underscore the significant burden of uncorrected refractive errors in special schools and the high prevalence of nystagmus in blind schools. The substantial proportion of avoidable visual impairment highlights the critical need for early screening and targeted interventions to improve visual function for children with disabilities.

## Introduction

Vision impairment has attracted widespread public concern with the increasing global population^1,2^. Globally, at least 2.2 billion individuals are affected by visual impairment^3^, contributing to substantial economic burdens^4^. In China, an estimated 2.03 million teenagers experienced moderate to severe vision impairment, and approximately 230 thousand teenagers were blind in 2019^2^. Visual impairment adversely impacts children’s academic performance, social development, and overall health throughout their lives^5^.

China has made notable progress in blindness prevention in recent years^2,6^. While numerous researches have explored the prevalence and causes of visual impairment, data specially focus on children with disabilities remain insufficient. According to a national statistical monitoring report, there were approximately 881,000 children with disabilities in China in 2020^7^. Evidence suggests that children with disabilities are more likely to experience visual impairment compared to their non-disabled peers^8,9^, yet they often face barriers to accessing eye health services^10^. This study, therefore, aims to evaluate the prevalence and causes of visual impairment among children with disabilities in special schools.

The prevalence and etiology of visual impairment exhibit regional variation^2^. Previous studies have demonstrated that geographic factors significantly influence the incidence of visual impairment^11,12^. Shanxi Province, an inland region where 80% of the area is mountainous and hilly, provides a unique setting for such research. Identifying the causes of visual impairment is crucial for governmental and institutional to provide precision aid. Accordingly, this study also investigates visual impairment among students in blind schools in Shanxi Province.

## Methods

This cross-sectional study was conducted between March and September 2018 across four cities in Shanxi Province. The study included two blind schools and nine special schools with two blind classes. Special schools serve children with various disabilities including intellectual disabilities, while blind schools and blind classes within special schools are designed specifically for students with total blindness or profound visual impairment. Ethics approval was obtained from the Ethics Committee of Shanxi Eye hospital (identifier SXYYLL-KSSC0017). All schools were government-funded and operated. Permission for the screening was obtained from school principals, and informed consent was secured from parents or guardians beforehand.

The examination team consisted of two ophthalmologists, two optometrists, and 10 nurses. Demographic data were collected, including age, gender, ethnicity, family history, urban/rural residence, prior systemic and ocular diseases, and history of ocular surgery. General health conditions, including hearing loss, mental retardation, physical handicap, epilepsy, and other related factors, were also recorded. For students absent from school, a mobile team—including an ophthalmologist, an optometrist, and a nurse, accompanied by a local teacher—conducted home visits to complete the examinations.

Distance visual acuity (VA) was assessed using the Single Lea Symbol Book Set (Good-Lite Company) at 3 m. For children with mental retardation who could not complete the test using the Single Lea Symbol Book Set, Cardiff Cards (Kay Pictures Ltd) were employed, and the results were converted to logMAR. Best-corrected VA was measured with trial lenses, or low-vision devices if already in use. Refractive error was initially determined without cycloplegia. However, cycloplegic refraction was conducted when the measured visual acuity fell below the age-specific normative threshold. Refractive status was determined through autorefraction (Topcon Corporation, Auto Kerato-Refractometers, KR-800), retinoscopy, and subjective correction. If subjective refraction was not feasible for children with mental retardation, best-corrected VA was evaluated based on retinoscopy. Pinhole VA was assessed when corrections were unsuccessful. Anterior segment examinations were performed with a handheld slit lamp (Kowa American Corporation, SL-19), while posterior segment evaluations were conducted using indirect ophthalmoscopy after pupil dilation with 1% tropicamide eye drops, whenever possible.

Data were recorded using WHO/PBL forms^13^. The causes and anatomical sites of visual impairment were identified, and visual impairment was categorized based on WHO classifications using best-corrected VA^3^. Spherical equivalent refraction was classified as emmetropia (− 0.50 D to + 0.50 D), myopia (< − 0.50 D), or hyperopia (> + 0.50 D). Spectacles were provided free of charge if corrections improved VA, and low-vision devices were distributed based on functional vision needs. Surgical requirements were documented, and referrals were made to nearby eye hospitals.

Statistical analyses were performed using SPSS version 26.0. Demographic data were summarized using descriptive statistics. Categorical variables were analyzed using frequencies and percentages, with group comparisons conducted via the Chi-square test.

## Results

### Special schools

A total of 441 children, with a mean age of 12.3 ± 3.5 years, were enrolled from nine special schools. Of these, 274 were male, and 167 were female. Among the 441 children, 387 (87.8%) had normal vision, while 44 (10.0%) exhibited vision impairment. This included 40 (9.1%) cases of mild visual impairment, 4 (0.9%) of moderate visual impairment. Another 10 (2.3%) cases were classified as undetermined or unspecified due to poor compliance (Table 1).

**Table 1:** WHO category of vision of the children in special schools.

	WHO category	Visual acuity (logMAR)	N	%
0	No visual impairment	VA ≤ 0.3	387	87.8
1	Mild impairment	0.5 ≤ VA < 0.3	40	9.1
2	Moderate visual impairment	1.0 ≤ VA < 0.5	4	0.9
9	Undetermined or unspecified		10	2.3
	Total		441	100

Uncorrected refractive error was the primary cause of vision impairment in 49 children (90.7%), while other causes included cataract, aphakia, and nystagmus (Table 2). Among the 387 children without vision impairment, uncorrected refractive error was identified in 280 children (72.4%).

**Table 2:** Ocular diseases and general health of children in special schools.

	Total	Ocular diseases		General health	
Visual impairment	54	Refractive error	49 (90.7%)	Mental retardation	42 (77.8%)
		Cataract	1 (8.4%)	Autism	5 (9.3%)
		Aphakia	1 (1.9%)	Hearing loss	4 (7.4%)
		Nystagmus	1 (1.9%)	General normal	3 (5.6%)
		Undetermined	2 (3.7%)		
			54 (100%)		54(100%)
No visual impairment	387	Refractive error	280 (72.4%)	Mental retardation	267 (69.0%)
		Normal	107 (27.6%)	Hearing loss	92 (23.8%)
				Autism	13 (3.4%)
				General normal	13 (3.4%)
				Others	2 (0.5%)
			387 (100%)		387 (100%)
Total	441				

Regarding general health conditions, 309 children (70.1%) had mental retardation, 96 (21.8%) had hearing loss, 18 (4.1%) had autism, and 16 (3.6%) were general normal but presented with ocular conditions such as strabismus, amblyopia, congenital cataract, nystagmus, or ptosis. Additionally, two children (0.5%) were diagnosed with attention deficit hyperactivity disorder and developmental disorders of scholastic skills, respectively. Subgroup proportions are detailed in Table 2.

### Blind schools and classes

A total of 56 children from blind schools and classes were included in the study, with ages ranging from 8 to 18 years (mean 13.0 ± 3.2 years). Among them, 33 (59%) were male and 23 (41%) were female. Two children were enrolled in schools but were homeschooled through teacher visits, while three children with combined visual impairment and multiple disabilities were identified during home visits; their data were excluded as their visual acuity could not be recorded per the study protocol.

The primary ocular causes of vision loss were nystagmus (25.0%), amblyopia (19.6%), and cataract (8.9%) (Table 3). Etiological analysis identified 25 cases (44.6%) of avoidable visual impairment (amblyopia, cataract, corneal leukoma, glaucoma, aphakia, retinopathy of prematurity) and 31 cases (55.4%) of unavoidable causes (nystagmus, retinal dystrophy, albinism, anophthalmia, cortical blindness, iris coloboma, microphthalmia, optic nerve atrophy). Among the 56 children, 10 resided in rural areas, and 46 were from urban settings. Avoidable blindness was observed in 9 of 10 rural children and 31 of 46 urban children, with no significant difference observed in the Chi-square test.

**Table 3:** Etiological causes of visual impairment categorized by ocular diseases.

Etiology	N	%
Nystagmus	14	25.0
Amblyopia	11	19.6
Cataract	5	8.9
Retinal dystrophy	5	8.9
Glaucoma	3	5.4
Aphakia	3	5.4
Optic nerve atrophy	3	5.4
Anophthalmia	2	3.6
Microphthalmia	2	3.6
Corneal leukoma	2	3.6
Iris coloboma	2	3.6
Cortical blindness	2	3.6
Albinism	1	1.8
Retinopathy of prematurity	1	1.8
Total	56	100

26 children (46.4%) had normal-appearing globes. Among those with ocular lesions, the most affected sites were the lens (8 children, 14.3%), retina (7 children, 12.5%), and the entire globe (7 children, 12.5%) (Table 4).

**Table 4:** Abnormal anatomical causes of visual impairment.

Anatomical site	N	%
Globe appears normal	26	46.4
Lens	8	14.3
Retina	7	12.5
Whole globe	7	12.5
Cornea	3	5.4
Optic nerve	2	3.6
Uvea	2	3.6
Others	1	1.8
Total	56	100

Based on WHO criteria for visual impairment, 30 children (53.6%) were classified as blind, 22 (39.3%) as visually impaired, and 4 (7.1%) as undetermined or unspecified. Specifically, 1 child (1.8%) had mild impairment (0.3 ≤ VA), 16 (28.6%) had moderate visual impairment (1.0 ≤ VA < 0.5), and 5 (8.9%) had severe visual impairment (1.3 ≤ VA < 1.0). Among the 30 blind children, 13 (23.2%) had blindness 4 (1.7 ≤ VA < 1.3), 15 (26.8%) had blindness 5 (PL ≤ VA < 1.7), and 2 (3.6%) had blindness 6 (NPL). VA of 4 blind children were undetermined or unspecified. Details are presented in Table 5.

**Table 5:** WHO category of vision of the children in blind schools and blind classes.

	WHO category	Visual acuity (logMAR)	N	%
1	Mild impairment	0.5 ≤ VA < 0.3	1	1.8
2	Moderate visual impairment	1.0 ≤ VA < 0.5	16	28.6
3	Severe visual impairment	1.3 ≤ VA < 1.0	5	8.9
4	Blindness	1.7 ≤ VA < 1.3	13	23.2
5	Blindness	PL ≤ VA < 1.7	15	26.8
6	Blindness	NPL	2	3.6
9	Undetermined or unspecified		4	7.1
	Total		56	100

*NPL* no perception of light, *PL* perception of light.

The onset of visual impairment was indeterminate in 30 children (53.6%) due to conditions such as congenital cataract, glaucoma or buphthalmos, retinoblastoma, and other birth abnormalities. Hereditary causes accounted for 14 cases (25%), postnatal causes (e.g., trauma and measles) for 7 cases (12.5%), and perinatal and intrauterine causes for 3 (5.4%) and 2 (3.6%) cases, respectively (Table 6).

**Table 6:** Etiological causes of visual impairment categorized by onset.

Etiology	N	%
Undetermined	30	53.6
Hereditary	14	25.0
Postnatal	7	12.5
Perinatal	3	5.4
Intrauterine	2	3.6
Total	56	100

## Discussion

On 8th October 2019, the WHO issued the first World Report on Vision^14^, highlighting that people with disabilities bear a heavier burden of vision impairment. Despite extensive research on the prevalence and causes of visual impairment in recent decades, studies rarely focus on children with disabilities, who often face significant barriers to obtaining adequate eye care. In this study, 10.0% (44 out of 441) of children in special schools were found to have visual impairment, primarily due to uncorrected refractive errors. Comprehensive eye examinations conducted in blind schools and classes further revealed that nystagmus was the leading cause of visual impairment. Importantly, visual impairment in 25 out of 56 children (44.6%) in blind schools and classes was deemed avoidable.

The prevalence of visual impairment in special schools was 10.0%. However, visual acuity assessments may have underestimated the severity of impairment in children with mental retardation, as these children often struggled with completing optotype tasks when symbols became smaller. Spectacles and low-vision devices were provided to children with hearing loss, which are expected to improve educational outcomes^15,16^. However, only spectacles were distributed to children with mental retardation due to the challenges in guiding their use of low-vision devices.

Notably, 10 children with visual impairment were assigned in special schools rather than blind schools or blind classes, which may have negatively impacted their daily activities and overall development. Moreover, 13 children in special schools had no systemic diseases or significant visual loss but presented with treatable conditions such as uncorrected refractive error, ptosis, or strabismus. These children could transition to regular schools upon appropriate treatment.

Over time, there have been notable changes in the primary causes of visual impairment^17^. A study conducted by Shi et al. across seven blind schools in China identified cataracts as the leading cause of visual impairment^18^. In contrast, the present study indicates that nystagmus is now the predominant cause. This shift likely reflects the influence of local policies that have facilitated early cataract detection and provided free treatment services. Children with improved visual acuity were integrated into regular schools, thereby excluding them from this study.

The etiology of nystagmus is complex and closely related to genetic factors, particularly in congenital nystagmus. Additionally, acquired nystagmus is closely associated with other diseases such as optic pathway diseases and central nervous system diseases. In this study, based on children’s medical history and on-site examination results, if nystagmus could be identified as a secondary symptom of an ocular disease (e.g., albinism), the etiology was not diagnosed as nystagmus. However, due to incomplete medical histories and limited examination conditions, some conditions (e.g., central nervous system disorders) could not be confirmed, and these children were diagnosed with nystagmus. Furthermore, visual impairment caused by various factors may also lead to nystagmus. In other words, for some children, nystagmus is the outcome rather than the cause of visual impairment. In conclusion, given the genetic factors and other diseases affecting visual input and eye movement associated with nystagmus, students in blind schools should undergo comprehensive ophthalmic examinations to identify potential underlying etiologies masked by nystagmus, thereby providing accurate and reliable references for policy formulation.

This study has some limitations. The widespread use of Cardiff Cards for children with mental retardation may complicate comparisons of visual impairment severity with standard logMAR assessments^19^. Additionally, functional vision, a critical factor for children with disabilities, was only evaluated when the use of low-vision aid devices was required, and the corresponding data were not systematically documented.

## Conclusions

In this study, the prevalence and causes of visual impairment among children with disabilities in Shanxi Province were evaluated. Among children in special schools, uncorrected refractive error was the primary cause of vision impairment, with most cases being preventable or treatable. However, some children with significant visual impairments were not appropriately assigned to blind schools or classes, potentially impacting their daily lives and development. In blind schools and classes, nystagmus emerged as the leading cause of visual impairment. Similarly, a substantial proportion of blindness cases could be preventable or treatable if detected early. Overall, the study revealed that numerous cases of vision impairment were avoidable, underscoring the importance of early screening and precision treatment.

## Data Availability

The datasets used and analyzed during the current study are not publicly available due to institutional policies but can be made available upon reasonable request. Interested researchers may contact the corresponding author for further information.
